# Whom to Believe? Understanding and Modeling Brain Activity in Source Credibility Evaluation

**DOI:** 10.3389/fninf.2020.607853

**Published:** 2020-12-14

**Authors:** Andrzej Kawiak, Grzegorz M. Wojcik, Piotr Schneider, Lukasz Kwasniewicz, Adam Wierzbicki

**Affiliations:** ^1^Chair of Neuroinformatics and Biomedical Engineering, Institute of Computer Science, Maria Curie-Sklodowska University, Lublin, Poland; ^2^Polish-Japanese Academy of Information Technology, Warsaw, Poland

**Keywords:** credibility, EEG, source localization, ERP, trust

## Abstract

Understanding how humans evaluate credibility is an important scientific question in the era of fake news. Source credibility is among the most important aspects of credibility evaluations. One of the most direct ways to understand source credibility is to use measurements of brain activity of humans performing credibility evaluations. Nevertheless, source credibility has never been investigated using such a method before. This article reports the results of an experiment during which we have measured brain activity during source credibility evaluation, using EEG. The experiment allowed for identification of brain areas that were active when a participant made positive or negative source credibility evaluations. Based on experimental data, we modeled and predicted human source credibility evaluations using EEG brain activity measurements with F1 score exceeding 0.7 (using 10-fold cross-validation).

## 1. Introduction

In 2020, the world has been fighting not only a pandemic, but more precisely—both a pandemic and an infodemic^1^. Spread of COVID-19 is accompanied by an equally unfortunate and dangerous spread of misinformation. One of the most striking examples is fake news that links COVID-19 epidemic to 5G technology^2^. Since 2018 fake news has been an active area of research (Lazer et al., 2018). At the same time, fake news has continued to spread through social media (such as Facebook or Twitter) (Allcott and Gentzkow, 2017). One of the main reasons is that messages on social media are forwarded based on trust that receivers have in their virtual friends (or trust that followers have in their Twitter sources). This makes it especially important to understand why social media users find messages from their virtual acquaintances so credible.

Unfortunately, we still lack knowledge of why people believe in fake news and why it spreads so easily. While social psychology has studied factors that might make receivers believe fake news more easily (Rutjens and Brandt, 2018; Forgas and Baumeister, 2019), these results are based on studies that rely on participants' declarations or on indirect inferences of their judgements from their behaviors. Simply asking participants whether they believe fake news, or inferring this conclusion from their behavior, is insufficient to conclude with certainty that fake news was found credible, nor can it reveal the real reasons for such a decision. In this article, we describe the results of a large experiment that aimed at understanding basic processes occurring in brain during credibility evaluation, by directly measuring brain activity. This is a new subject in neuroinformatics and neuroscience, as most research that used EEG or fMRI in the context of credibility has focused on lie detection (Wang et al., 2016; Meijer and Verschuere, 2017), which is based on the investigation of the brain activity of the author, and not the receiver of the message.

In the future, we envisage the use of EEG for testing the credibility of information that may either be fake news, or (to the contrary) correcting information designed to counteract fake news. Similarly to the use of EEG in online marketing (Deitz et al., 2016; Guixeres et al., 2017), in such a setting researchers could evaluate the credibility of information for a panel of information consumers.

The main goal of our research is to describe and understand brain activity during credibility evaluation. In this article, we focus on the aspect of credibility that is related to the source of the message: source credibility. Source credibility is especially important in the situation when social media users receive fake news outside of their area of expertise and experience (which is typical of many fake news, like the quoted example of COVID-19 and 5G). Our goal is to identify brain areas and periods of brain activity that are most active and most important in the process of source credibility evaluation. This basic question leads to a more applicable goal: creation of a method for EEG-based source credibility evaluation that would work only on the basis of observed brain activity.

## 2. Related Work

### 2.1. Source, Message, Media Credibility

The concept of credibility, similarly to the concept of trust, is grounded both in science and in common sense. Credibility has been subject to research by scientists, especially in the field of psychology and media science. One of the earliest theoretical works on credibility dates back to the 1950s. This influential work of the psychologist Carl Hovland (Hovland and Weiss, 1951) introduced the distinction between ***source, message, and media*** ***credibility***. Out of these three, two are a good starting point for a top-down study of the complex concept of credibility: source credibility and message credibility. These two concepts are closely related to the natural-language (dictionary) definitions of the term “credibility.” In the English language dictionary (Oxford Advanced Learner's Dictionary), credibility is defined as “the quality that somebody/something has that makes people believe or trust them.” When this definition is applied to a person (“somebody”), it closely approximates source credibility—an essential concept in real-life, face-to-face communication. However, it should be noticed that the dictionary definition of credibility can also be applied to “something”—the message itself. And, in many online environments, message credibility must be evaluated without the knowledge about the source.

Credibility has also been the subject of research in social psychology (Rutjens and Brandt, 2018; Forgas and Baumeister, 2019). Several psychological factors that affect credibility evaluations have been identified. Belief in conspiracy theories and fake news is, for instance, correlated with radical political leanings, as well as being affected by confirmation biases, group polarization, overconfidence, and statistical illiteracy (Forgas and Baumeister, 2019). Social psychology has also evaluated the effectiveness of various interventions aiming to correct misinformation. Research has found, for example, that a simple denial or contradiction is much less effective than repeated statements of true information (Forgas and Baumeister, 2019). In principle, solid empirical results from social psychology can be confirmed by EEG or fMRI analysis of brain activity. In a recent article (Moravec et al., 2018), researchers used EEG to measure and confirm the effect of confirmation biases on credibility evaluation. However, research investigating selected psychological factors should be based on good understanding of the basic activities that occur in the brain during credibility evaluation. The goal of our research is to study such basic brain activities through experiments controlling and limiting factors that can influence credibility evaluations.

Information scientists have studied credibility evaluations with the goal of designing systems that could evaluate Web content credibility automatically or support human experts in making credibility evaluations (Wawer et al., 2014; Liu et al., 2015; Kakol et al., 2017). However, human credibility evaluations are often subjective, biased or otherwise unreliable (Kakol et al., 2013; Rafalak et al., 2014), making it necessary to search for new methods of credibility evaluation, such as the EEG-based methods proposed in this article.

### 2.2. Source Credibility

Search for the term “source credibility” on Google Scholar returns an excess of 12,000 results (for an overview of recent publications, especially on the subject of Web content credibility, see Wierzbicki, 2018). Research in this subject has ranged from investigating impact of source credibility on politics (Flanagin and Metzger, 2017) to healthcare (Kareklas et al., 2015).

Previous theoretical research established that *source credibility is closely related to credibility trust* (Wierzbicki, 2018). It results from an expectation that the source would observe social norm of not lying (not communicating a false message). Following the analogy to trust, source credibility can also be based on the trustworthiness of the source in the context of veracity; it is difficult, however, to reliably observe, measure or predict this property. Most observations or valuations concerning credibility are done in a relational setting: communication of a *message* from a *source* to a *receiver*. A proxy for credibility trustworthiness may be *source reputation* in the context of veracity, estimated based on the past performance of the source. Therefore, it can be concluded that *source credibility is a combination (or multiple criteria evaluation) of two kinds of trust: credibility trust and the trust in the expertise of the source*. These two types of trust are independent and complementary; a source might, after all, usually tell the truth, but not be able to do so because of lack of expertise in a given subject. On the other hand, an expert in the subject may not be trustworthy due to the fact of being a habitual liar.

### 2.3. Experimental fMRI and PET Findings

So far, there has been little research reported in the scientific literature that directly attempted to investigate brain activity involved in credibility evaluation. While there has been a great amount of research on lie detection (Wang et al., 2016; Meijer and Verschuere, 2017), this is a topic that investigates brain activity of the sender, not of the receiver of unreliable information. Not much has been done in the field of source credibility research as far as neuroimaging methods are concerned. Source credibility is associated with trustworthiness, which was discussed, for example, by Rosenbloom et al. (2012). They state that usually the amygdala is involved in trusting others (Rosenbloom et al., 2012).

There were some attempts to investigate phenomena that are not equivalent to credibility evaluation, but are in some way associated with cognitive processing that is related to credibility. These phenomena were related to decision making under uncertainty and to moral judgments. In both cases, the correctness of the decision was related to the task of credibility evaluation. The subjects needed, in essence, to evaluate the credibility of the following statements: “this is the correct choice given the possible uncertain outcomes” or “this is the right choice in a moral dilemma.”

In most of the reported experiments, participants were asked to make decisions under uncertainty. One of the works that is most important and significant for our research is presented in Stern et al. (2010), where subjects were updating their knowledge needed to make decisions. Uncertainty was updated task by task, leaving less space for subjective choice. The increased activity in Brodmann Areas (BA) 32, 34, 9, 10, and 32 was reported in their fMRI study. From the perspective of our research, the most important is BA 10, which corresponds to the Orbital gyri in the Orbital cortex of the frontal lobe.

Orbital gyri covers also BA 11, which together with BA 10 was observed to be hyperactive in the experiment presented in Rogers et al. (1999). In that experiment, which used PET, participants were asked to choose between a small, more likely reward and a large one that was unlikely.

BA 24 as well as left cingulate gyrus and sulcus seem to play an important role in decision-making process where some moral choices are intended to be undertaken by participants (Luo et al., 2006) as well as in Stern et al. (2010) again in fMRI experiments.

Note that emotional engagement and moral dilemmas involve the activity of Ba 9, 10, and 24, as reported in a few fMRI studies (Greene et al., 2001, 2004, 2008).

The likely and unlikely rewards are in some way variations of Iowa Gambling Tasks, and they were also investigated using fMRI toward finding gender-related differences (Bolla et al., 2004) as well as, for example, in Fukui et al. (2005) where risk anticipation was measured and the hyperactivity of medial Frontal Gyrus on the border of BA9 and BA 10 was noted in 3 T fMRI scanner.

Again, the deficit of moral judgements was observed in Ciaramelli et al. (2007) in fMRI investigated subjects with damaged ventromedial prefrontal cortex.

One of the most interesting fMRI experiments was discussed in Schaich Borg et al. (2006) where actions, decisions, intentions and consequences were investigated together with moral dilemmas. Intended harm and unintended harm were distinguished, and it was found that different brain systems are involved in decision making with harmful consequences. In all cases the hyperactivity of BA 10, 11, and 12 was observed.

## 3. Experiment Design

The aim of the experiment was to observe activity of the participant's brain cortex during performance of a task involving source credibility evaluation. In order to ensure that the participants could rely only on source credibility during the experiment, the experiment was designed so that the participants would not be familiar with the topics of the messages. The selected message topics concerned a Japanese language test.

The experiment described in this article was preceded by a pilot experiment conducted a few months earlier at the same laboratory, involving two times fewer participants (57). The goal of the pilot experiment was to test the experiment design. The experiment described in this article had an improved design based on the results of the pilot experiment (Kawiak et al., 2020).

### 3.1. Participants and Ethical Commission's Permission

In order to simplify EEG measurement, all participants selected for the experiment were right-handed males. A total of 111 participants took part in the experiment. *All experiment participants had no knowledge of Japanese. The Japanese language is not popular in Poland, it is not taught neither in schools nor in our University. Students before the participation filled up the questionnaire in which they were asked to tick the appropriate level of knowledge of foreign languages as well as their age and others*.

The experiment was carried out at Marie Curie Skłodowska University in Lublin, Poland, between October, 15th and December, 15th, 2019. All participants were university students, and therefore they were of 21–22 years of age. They were recruited by and received a reward for participation in the experiment in the form of course points The experiment was carried upon the permission of the Universtity's Bioethical Commission (MCSU Bioethical Commission permission 13.06.2019).

### 3.2. Source Credibility Evaluation Task

In the introduction to the experiment, participants were informed that fictitious “students” of another university had solved a test regarding their knowledge of Japanese Kanji signs (after one semester of learning completed), and that we know the results achieved by all students.

For our experiment, student names were chosen at random. The fictitious students were divided into three groups—those who received 50, 70, and 90% of the maximum score to be gained during the test. We believe that there is no much sense to evaluate results of fictitious students when one knows about their less their <50% performance which means that in most of cases such student is wrong.

**Figure 1:**
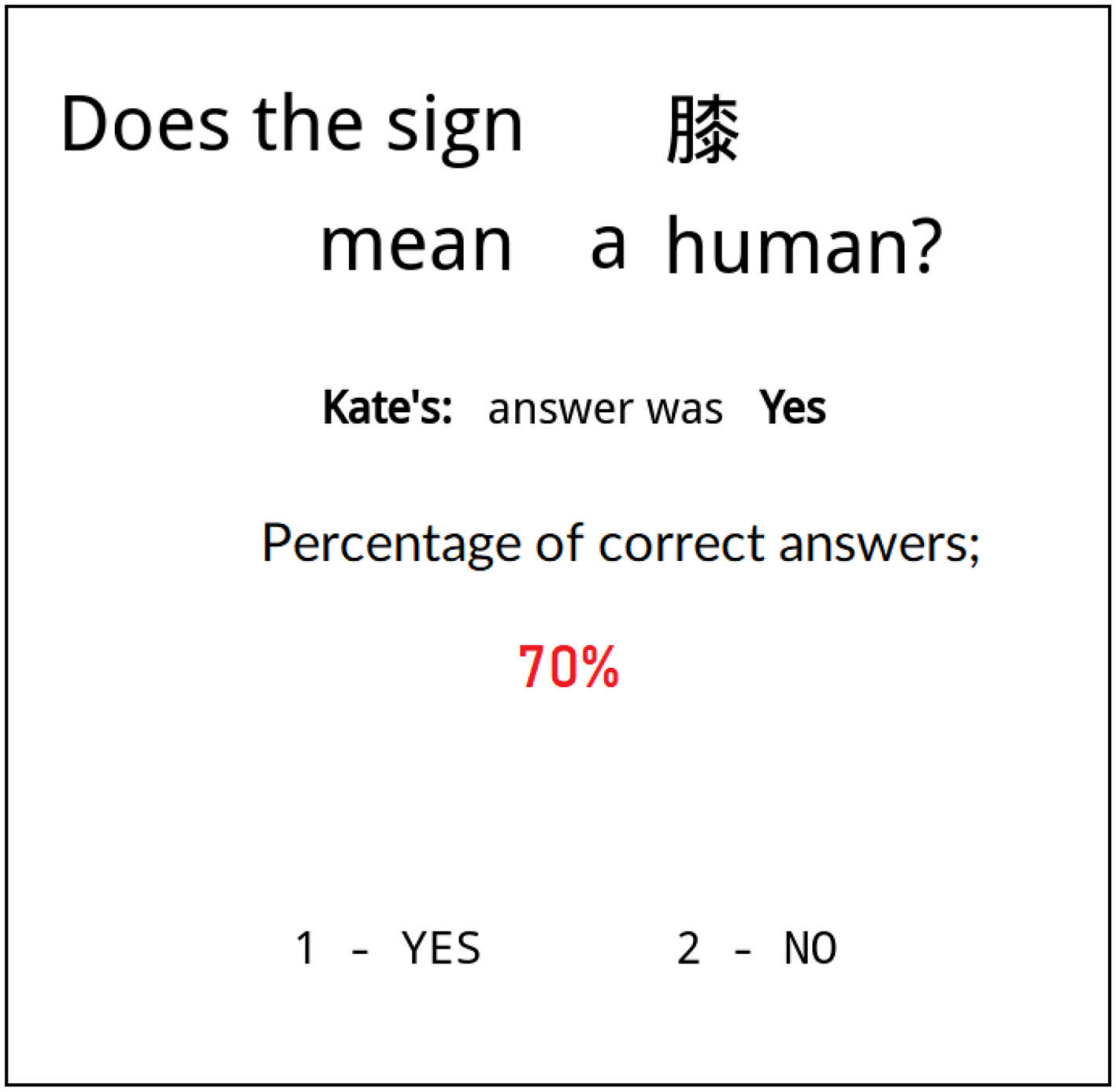
Typical screen shown to a participant during the experiment. Student's name, hint and hinting student's accuracy during the entire test are in the center of the screen. Participant was asked to agree or disagree with the student's answer (bottom) based on their credibility evaluation of the hint that was influenced by the student's accuracy. The names of students (here Kate) were chosen to be the most popular names of students in Poland with assumption that thanks to it there will be no significant influence of the name on participant's decision.

Experiment participants were shown 180 screens with one Kanji sign on each of them and with the question whether the translation of that sign was correct or not. As a hint, participants received information from one of the “students” (represented by their name) who had an overall accuracy of 50, 70, or 90% during the test. The hint was the student's answer (“Yes” or “No”) to the question posed to the participant (see Figure 1). The same accuracy of the hinting student (with SCL 50, 70, or 90%) was shown on 60 (out of 180) screens.

Note that the participants did not know whether the student's response was correct or incorrect. The only thing a participant knew was the student's result regarding the entire test. In this way, we have created a situation in which the participant had to make a decision whether to accept a message (the student's hint) based on source credibility (the student's overall score in the test).

In the experiment, the participants faced a binary decision: they were asked to press a “YES” or “NO” button. This decision could mean agreeing with the student's hint, in which case we shall refer to this decision as “trusting.” The participants could also disagree with the student's hint, in which case we shall refer to it as “distrust.” Note that both trusting and distrusting decisions can be “YES” or “NO,” but this is not relevant to the experiment. The only relevant aspect of the participant's decision is whether it was trusting or distrusting, corresponding to a positive or negative source credibility evaluation, respectively.

Recall that source credibility can be thought of as the source's reputation in the context of expertise or veracity. In our experiment, the only information that participants had about the students' reputation was the test score. If the participants were informed that the suggesting student's test score was 90%, they would probably make a trusting decision. If the test score was 50%, we could expect that the participant would respond randomly. We shall refer to the hinting student's test score as the Source Credibility Level (SCL).

Moreover, the participants were not given the correct meaning of the current and previous signs presented to them. Thus, the participants were not rewarded for a good answer and were not punished for a bad one. This experiment design ensured that participants made decisions in a non-competitive setting and without consideration for a reward.

Repeating similar screens 60 times for each source credibility level made it possible to observe the so-called Event-Related Potentials (ERPs) in the electroencephalographic activity registered by the amplifier in our lab. The methodology of ERP is probably most often used in experimental psychology, and observations made using source localization methods allowed us to measure brain cortex activity quantitatively.

### 3.3. Experimental Cases and Data

All decisions made by experiment participants can be classified into the following six cases that allow us to compare brain activity for trusting and distrusting decisions under stimulus of various source credibility levels.

Additionally, let us introduce three larger sets of all decisions made while the participant was shown a particular source credibility level: *A*_50_ = *T*_50_ ∪ *D*_50_, *A*_70_ = *T*_70_ ∪ *D*_70_, and *A*_90_ = *T*_90_ ∪ *D*_90_, and two sets of all trusting and all distrusting decisions, regardless of the SCL: *T* = *T*_50_ ∪ *T*_70_ ∪ *T*_90_ and *D* = *D*_50_ ∪ *D*_70_ ∪ *D*_90_.

### 3.4. EEG Measurements

Our empirical experiments involved top EEG devices. We were equipped with a dense array amplifier recording the cortical activity with up to 500 Hz frequency through 256 channels HydroCel GSN 130 Geodesic Sensor Nets provided by EGI^3^. In addition, in the EEG Laboratory the Geodesic Photogrammetry System (GPS) was used. The artifact detection and elimination was proceeded by standard scripts provided by the EGI under the manual supervision of lab workers.

Estimating ERP for each of the 256 electrodes is not necessary for ERP observation, as in general standards there are just a few electrodes (in our case 26) playing an important role in cognitive tasks^4^. Therefore, at the beginning the raw EEG time series were post-processed, averaged and ERPs were estimated for 26 cognitive electrodes. In the following discussion, when we refer to differences in the cognitive ERP signal, it means that in a certain time interval the ERP signal averaged over 26 cognitive electrodes was different.

However, for the sLORETA source localization analyses (used for verification of the next hypotheses) the ERP from all 256 electrodes had to be in fact calculated on the fly. Here, it should be pointed out that ERP and activity measures based on source localization are two different measures. ERP can be calculated for each electrode (although we have considered only 26 cognitive electrodes). However, the activity (MEC) of particular Brodmann Areas was calculated using all 256 electrodes, making the resolution sufficient to achieve high accuracy.

Having the ERP signal estimated for each electrode out of 256, it was possible to calculate the mean electric charge (MEC) flowing through the BA situated under these electrodes on the brain cortex in CPTR. Moreover, it was also possible to conduct the full source localization analysis of the signal originating from all 256 electrodes using sLORETA algorithm (GeoSourse parameters set as follow: Dipole Set: 2 mm Atlas Man, Dense: 2,447 dipoles Source Montages: BAs). Mean electric current flowing through each BA and varying in time was given as an output. Having those values calculated, it was possible to integrate that current in time and then get the MEC. The mean electric charge calculated for each electrode using source localization techniques could, as we intended, indicate the hyperactivity of some BAs that are not necessary precisely situated under the cognitive electrodes. For all calculations of MEC the CPTR was divided into 10 ms time intervals. The procedure of calculating MEC has been described in detail in Wojcik et al. (2018).

We shall denote the MEC by $\mu_{b}^{\left(t_{1},t_{2}\right)}$, where *b* ∈ {1, …, 148} is the index of the brain area, while (*t*_1_, *t*_2_) is the time interval. Note that *t*_2_ − *t*_1_ ≥ 10*ms*, but we can also calculate the MEC in longer time intervals. Note also that we calculate the MEC based on ERP signals calculated from a subset of participant decisions—usually from a subset of identical decisions (to trust or to distrust the source). Therefore, the variables $\mu_{b}^{\left(t_{1},t_{2}\right)}$ can be used as independent variables related to a single participant's decision.

### 3.5. Experiment Hypotheses

Our experiment had been designed to limit the stimulus received by participants to source credibility. Participants had to make a binary credibility evaluation. Therefore, our first hypothesis concerns the relationship between the SCL and number of trusting decisions.

1. An increase of SCL leads to a significant increase in the number of trusting decisions of participants.

To verify hypothesis 1, it is sufficient to conduct a statistical test to compare the number of trusting decisions for the cases *A*_50_, *A*_70_, and *A*_90_. Hypothesis 1 is also a test of our experiment's internal validity. As SCL is the only stimulus in our experiment, and this stimulus preceded the participant's decision in time, to test internal validity it should be verified whether or not the stimulus (SCL) and the decision (trusting or distrusting) varied together.

Further hypotheses concern differences in observed cortical brain activity for varying levels of SCL and for trusting or distrusting decisions. Specifically, we shall compare average amplitudes of ERP signals from all 26 cognitive processing electrodes during time intervals within 0–800 ms from stimulus. In short, we shall refer to the ERP signals averaged from all cognitive processing electrodes as *cognitive ERP signals*. We made the following hypotheses:

2. the cognitive ERP signals in a certain time interval have statistically significant differences for different source credibility levels of 50, 70, and 90% (in cases *A*_50_, *A*_70_, *A*_90_).
3. the cognitive ERP signals in a certain time interval have statistically significant differences for pairs of cases with different levels of SCL: *T*_50_ and *T*_90_, *T*_70_ and *T*_90_, *T*_50_ and *T*_70_; *D*_50_ and *D*_90_, *D*_70_ and *D*_90_, *D*_50_ and *D*_70_.
4. the cognitive ERP signals in a certain time interval have statistically significant differences for trusting and distrusting decisions, in cases: *T* and *D*, *T*_90_ and *D*_90_, *T*_70_ and *D*_70_.

Hypotheses 2 and 3 regard the effect of SCL on cognitive ERP signals. The effect is considered jointly for all participant decisions in 2, and separately for trusting and distrusting decisions in 3. Hypothesis 4 concerns the differences in cognitive ERP signals between trusting and distrusting decisions, for various levels of SCL and independently of SCL.

When verifying hypotheses 2, 3, and 4, we will investigate consecutive time intervals of 10ms within the cognitive decision making time interval. We shall use statistical tests to compare cognitive ERP signals from various experiment cases within a selected time interval. Then, we shall select the longest joint time interval during which hypothesis holds. The comparison of these time intervals for the various hypotheses brings additional insight into the analysis.

Next hypothesis concerns mean electric charge (MEC) flowing through all Brodmann Areas (BAs). We have used these measurements to consider the third research question: whether it is possible to model and predict source credibility evaluations using EEG measurements.

5. mean electric charge flowing through various brain areas is sufficient to predict the decision to trust or distrust during the experiment, with an accuracy that significantly exceeds the baseline.

Note that hypothesis 5 concerns the possibility of creating an EEG-based method of source credibility measurement. While this is only a first step, a positive validation of hypothesis 5 would open an avenue of investigating EEG-based source credibility measurements in other, more complex and realistic scenarios. Note that the baseline accuracy for hypothesis 5 is 50% (experiment participants make binary decisions).

### 3.6. Statistical Testing Methodology

For all statistical tests used to verify hypotheses 1–4, we will use the Mann-Whitney-Wilcoxon test, since the data distributions are not normal. We shall consider that statistically significant differences in the average ERP signals exist if the test is positive with a *p*-value of at most 0.05. Note that, since we are testing the same hypothesis, but in different time intervals, we do not need to correct the statistical significance for repeated testing.

## 4. Experiment Results

In this section, we report the results of our experiment, and verify the experimental hypotheses. We start with hypothesis 1 that concerns the impact of the Source Credibility Level (SCL) on participants' trusting or distrusting decisions. Next, we describe the measured brain activity, and consider hypotheses 2–4 that hypothesize the existence of statistically significant differences between cognitive ERP signals obtained from various experimental cases—combinations of SCL levels and trusting or distrusting decisions. Finally, we report the results of modeling the participants' decisions using mean electric charge flowing through various brain areas. The accuracy of this model is used to verify hypothesis 5.

### 4.1. Impact of Source Credibility Level on Trusting and Distrusting Decisions

**Figure 2:**
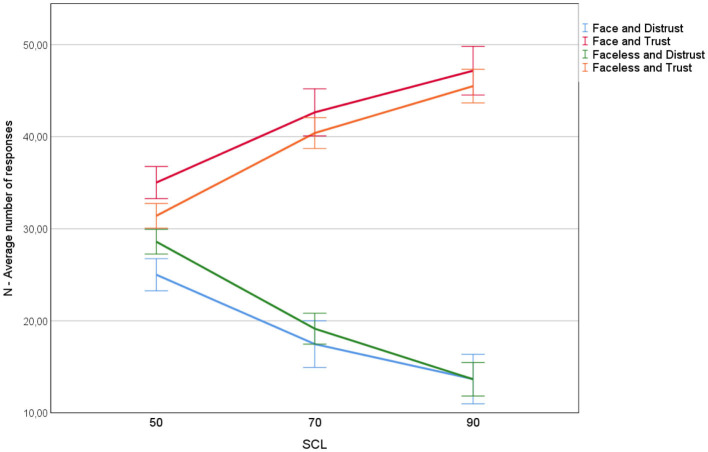
The number of trusting and distrusting responses as a function of SCL and presence or absence of the face avatar.

Verification of 1 is based on a simple count of the number of trusting decisions of all experiment participants for the three different levels of SCL: 50, 70, and 90%. This relationship is plotted on Figure 2. The figure also shows the results from the earlier pilot experiment, during which participants were presented with screens similar to Figure 1, but additionally displaying the hinting student's avatar (Kawiak et al., 2020). The avatar was a face randomly generated using a repository available at www.makeavatar.com and a simple Python script. The avatars had neutral emotions obtained by turning the smile-option off.

As shown on Figure 2, there exists a strong and statistically significant relationship between SCL and the number of trusting and distrusting decisions. Increasing SCL increases the number of trusting decisions, and decreases the number of distrusting decisions. The same relationship has been observed in the main experiment and in the pilot experiment with face avatars. However, the presence of a face increased the number of trusting decisions and decreased the number of distrusting decisions. This observation is consistent with the results of previous research on the relationship between the existence of profile pictures and user ratings (Xu, 2014).

Observation regarding the increase of the number of trusting decisions along with the increase of SCL positively verifies hypotheses 1. This result also confirms the internal validity of our experimental design, as we are able to observe significant differences in source credibility evaluations (trusting or distrusting decisions) for various levels of the stimulus (SCL).

### 4.2. Brain Activity Measurements

We proceed now to the investigation of brain activity in the various experimental settings. Recall that we have defined 6 basic experimental cases (Table 1). *T*_50_, *D*_50_, *T*_70_, *D*_70_, *T*_90_, and *D*_90_ (see section 3.3). These cases correspond to combinations of three levels of SCL (50, 70, and 90%) and trusting or distrusting participant decisions. The experimental hypotheses 2–4 (see section 3.5) concern the existence of statistically significant differences in ERP signals for pairs of experimental cases, or their combinations.

**Table 1:** Six experimental cases corresponding to various source credibility levels (SCL) and trusting or distrusting decisions.

**Case**	**SCL (%)**	**Participant's decision**
*T*_50_	50	Trusting
*D*_50_	50	Distrusting
*T*_70_	70	Trusting
*D*_70_	70	Distrusting
*T*_90_	90	Trusting
*D*_90_	90	Distrusting

Recall that the ERP signal has been calculated for 26 cognitive electrodes (see section 3.4), and the hypotheses concern statistically significant differences in the average ERP signal from the cognitive electrodes within a certain time interval. The time interval is being chosen successively investigating short time intervals of 10 ms. In each of these short intervals, we compare ERP signals averaged from the two experimental cases chosen for the comparison. We use the Mann-Whitney-Wilcoxon with a *p*-value of 0.01 to verify whether the cognitive ERP signals from two cases have statistically significant differences. We attempt to find the longest joint time interval (consisting of consecutive 10ms long time intervals) during which a hypothesis holds.

**Figure 3:**
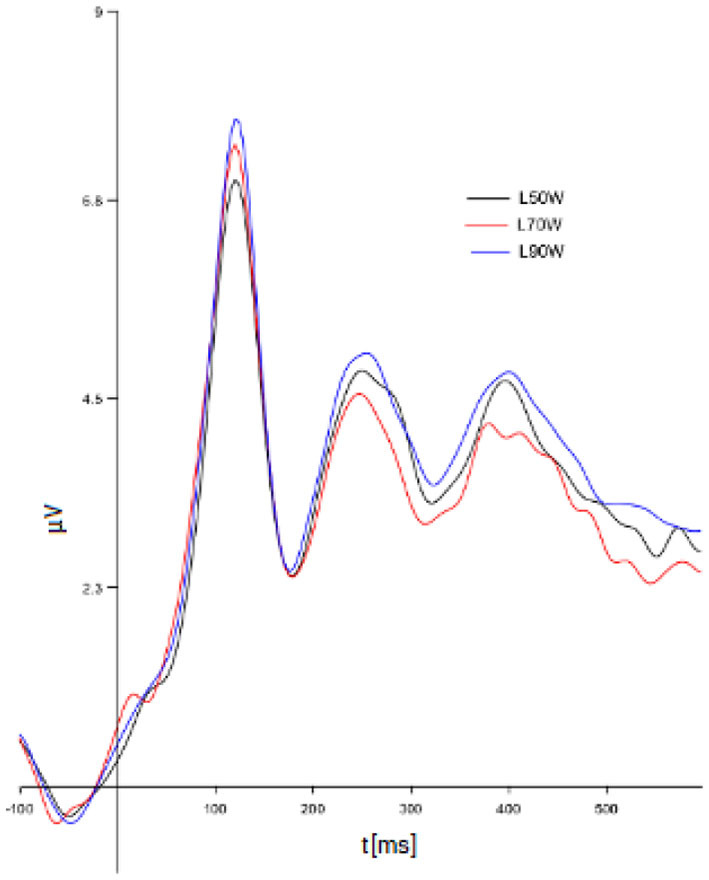
ERP signals averaged from cases: *A*_50_, *A*_70_, and *A*_90_ (for all decisions and different Source Credibility Levels).

The cognitive ERP signal averaged from the experimental cases *A*_50_, *A*_70_, and *A*_90_ has statistically significant differences in the following time intervals: 268–300 ms (*p* = 0.00833), 336–352 ms (*p* = 0.0068), 372–796 ms (*p* = 0.0009). This positively verifies the hypothesis 2 (see Figure 3).

The research like this the earliest time intervals always start at about 250 ms as earlier there is not conscious cognitive processing in the brain.

The significantly different time intervals are not continuous as we considered particular parts of the time in which the difference is observed, not the total continuous range of processing. If the total range of processing had been considered too many features would be averaged and no significant difference could have been observed at all.

Also, there are statistically significant differences observed for comparisons of all other pairs of experimental cases from hypothesis 3.

Statistically significant difference for the comparison of the experimental cases *T*_50_ and *T*_90_ (trusting decisions made under stimulus SCL = 50% and SCL = 90%) was observed in the following time intervals: 456–516 ms (*p* = 0.005), 544–640 ms (*p* = 0.0095), 672–760 ms (*p* = 0.0099). For distrusting decisions under the same stimuli (cases *D*_50_ and *D*_90_), significant differences in the EEG signal occurred in the 416–796 ms time interval (*p* = 0.0031). Note that the experimental case *D*_90_ has much fewer observations than other experimental cases, due to the decreasing number of distrusting decisions with increasing SCL (see Figure 2).

Statistically significant differences in the EEG signal were observed for a comparison of the cases *T*_70_ and *T*_90_ in a shorter time interval than for the cases *T*_50_ and *T*_90_: 456–516 ms (*p* = 0.005), 544–640 ms (*p* = 0.00964) from stimulus. This means that in the time interval 672–760 ms, significant differences in ERP signals from trusting decisions occur only for larger differences in SCL (SCL = 50 vs. 90%). On the other hand, the interval of significant difference between ERP signal for cases *D*_70_ and *D*_90_ was the same as for the cases of *D*_50_ and *D*_90_: 416–796 ms.

Finally, comparing the cases *T*_50_ and *T*_70_, we found statistically significant differences in ERP signals for a large time interval: 260–304 ms (*p* = 0.0082), 336–356 ms (*p* = 0.0056), and 440–796 ms (*p* = 0.0047). Note that the last interval includes the time intervals for which significant differences were observed between pairs of cases *T*_50_ and *T*_90_, as well as *T*_70_ and *T*_90_. A comparison of cases *D*_50_ and *D*_70_ revealed significant differences in ERP signals in the 376–796 ms (*p* = 0.0023) time interval. Again, this is a broader time interval than for pairs of cases *D*_50_ and *D*_90_, as well as *D*_70_ and *D*_90_. We conclude that significant differences in ERP signals occur for the change of the stimulus from SCL = 50% to SCL = 70%.

The above analysis confirms the hypothesis 3. Statistically significant differences in ERP signals have been observed for all pairs of basic cases, separately for trusting and distrusting decisions. We now turn to a comparison of ERP signals for trusting and distrusting decisions.

**Figure 4:**
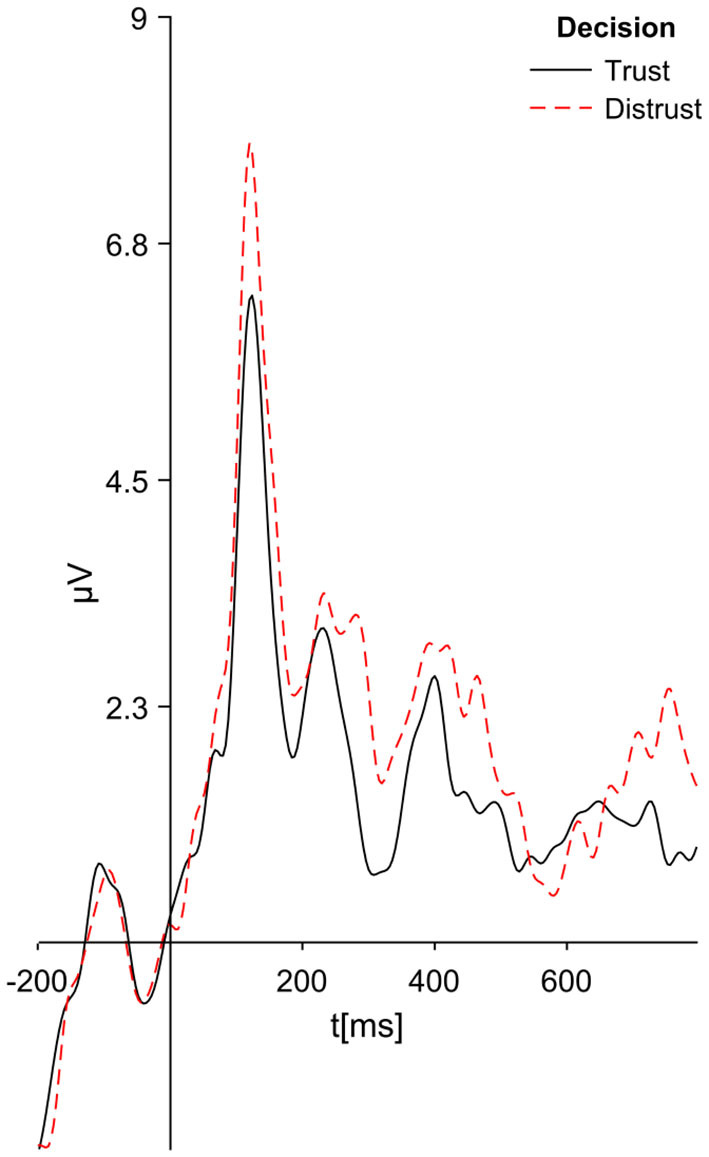
ERP signals averaged from cases *T* and *D* (for trusting decisions distrusting decisions separately, for all Source Credibility Levels).

Recall that hypothesis 4 concerns differences in ERP signals obtained from pairs of experimental cases: *T*_90_ and *D*_90_; *T*_70_ and *D*_70_, as well as the case *T* = *T*_50_ ∪ *T*_70_ ∪ *T*_90_ and *D* = *D*_50_ ∪ *D*_70_ ∪ *D*_90_. We have found statistically significant difference in ERP signals in all pairs of cases. The differences in cases *T* and *D* are the most interesting (see Figure 4). We have found significant differences in ERP signals in two broad time intervals: 268–300 ms (*p* = 0.0076) and 372–796 ms (*p* = 0.0007) from stimulus. This observation justifies the conclusion that brain activity is significantly different for trusting and distrusting decisions. It should be noted that the differences in the ERP signals occur also after 1 s from the stimulus—this is the time interval where conscious decisions are made^5^. We shall use the time intervals of significant differences between cases *T* and *D* as a starting point to aggregated Mean Electric Charges, to create models that will learn and predict positive and negative source credibility evaluations (trusting and distrusting decisions).

One additional case ought to be taken into consideration at this point: *T*_50_ and *D*_50_. Participant when offered such choice had random situation and according to our expectations there were no significant difference observed for such case.

Cognitive ERP signals also have significant differences for pairs of cases: *T*_90_ and *D*_90_ in the time intervals 316–352 ms (*p* = 0.091) and 624–784 ms (*p* = 0.0069), and *T*_70_ and *D*_70_ in time intervals: 248–272 ms (*p* = 0.0098) and 600–796 ms (*p* = 0.0094). These differences positively validate hypothesis 4.

We would like to inform the readers that in order to recognize statistically significant difference in the all of abovementioned cases the *p*-value returning from our statistical analysis had to be lesser or equal 0.01 (*p* < 0.01).

### 4.3. Machine Classification Models of Source Credibility Evaluations

Recall that 111 participants completed the experiment. They responded to questions that had different levels of SCL: 50, 70, and 90%. The responses of each participant to questions with different levels of SCL were treated as separate observations. We have averaged the ERP signal for trusting and for distrusting responses of a single participant for a fixed value of SCL. Based on this, we have calculated the Mean Electric Charge (MEC, see section 3.4) in each of the 148 brain areas and in every time interval of 10 ms. Recall that MEC from adjacent time intervals can be added to form MEC from a longer time interval. We denoted MEC by $\mu_{b}^{\left(t_{1},t_{2}\right)}$, where *b* is the index of the brain area, and (*t*_1_, *t*_2_) is the time interval. The explanatory variables of our models are MEC values from the same time interval and from all 148 areas of the brain. We have tested models for various time intervals, but have obtained best results for explanatory variables equal to $\mu_{b}^{\left(500,750\right)}$.

The SLC of 50% served as a baseline for observing changes in brain activity when the SCL was increased to 70 or 90%. Therefore, observations for *SCL* = 50% were not included in the training of machine classification models of participants' decisions. We split the dataset randomly into the *training set (79% of observations, from 88 participants for 2 different SCL levels and for 2 decisions: trusting or distrusting, which gives 352 observations)* and the *testing set (the remaining 21%, from 22 participants for 2 different SCL levels and for 2 decisions, which gives 88 observations)*. We have also used bootstrapping to obtain more accurate models.

We have first used Logistic Regression to train a classifier of positive or negative source credibility evaluations (trusting/distrusting decisions of participants). We chose this method to create an explainable model that would give greater insight into the brain activities during source credibility evaluation.

The first logistic regression model used all 148 brain areas as explanatory (independent) variables. We have used bootstrapping (10,000 times) to determine regression coefficients and their confidence intervals. The confusion matrix of this model is shown on Table 2. The logistic regression model achieved an accuracy of 75.0%, with *F*1 = 0.7576, *Recall* = 0.7409 and *Precision* = 0.7750 (see Table 3). This is a very satisfying result that positively validates Hypothesis 5: MEC values can indeed be used to predict participants' source credibility evaluations with high accuracy. It should be also noted that the model is very stable for different random splits of the data into training/testing sets.

**Table 2:** Confusion matrix of logistic regression model for predicting Source Credibility Evaluations, using all 148 brain areas.

	**Negative**	**Positive**
	**(Distrusting) (%)**	**(Trusting) (%)**
Predicted negative	77.5	22.5
Predicted positive	27.1	72.9

Model has been prepared using bootstrapping (10,000 times). The table shows the rates of true negatives, false negatives, false positives, and true positives.

**Table 3:** Quality measures of logistic regression models for predicting source credibility evaluations, using all 148 brain areas (full model) or selected 28 brain areas.

	**Full model (148 variables)**	**Model with 28 variables**
Accuracy	0.7520	0.7
F1 Measure	0.7576	0.7018
Precision	0.7750	0.7060
Recall	0.7409	0.6976
AUC	0.70	0.6

*Models have been prepared using bootstrapping (10,000 times)*.

The second model used a reduced number of explanatory variables. Our goal in building this model had been to identify the brain areas that were most significant for predicting the decision about source credibility evaluation. We have chosen 28 brain areas that had values of the logistic regression beta coefficient higher than the median of all 148 explanatory variables in the first model. In other words, the second model used a subset of the variables of the first model that had the highest beta coefficients. Similarly to the first model, we have used bootstrapping (10,000 times) to determine regression coefficients. The confusion matrix of the second model is shown in Table 4. The second model achieved a lower recall, but a higher precision as compared to the first, full model. The second model's overall accuracy, F-measure and AUC are similar to the full model, thus confirming that the chosen subset of variables is sufficient for modeling the experiment participants' decisions (see Table 3).

**Table 4:** Confusion matrix of logistic regression model for predicting Source Credibility Evaluations, using 28 brain areas.

	**Negative**	**Positive**
	**(Distrusting) (%)**	**(Trusting) (%)**
Predicted negative	70.6	29.4
Predicted positive	30.6	69.4

Model has been prepared using bootstrapping (10,000 times). The table shows the rates of true negatives, false negatives, false positives, and true positives.

### 4.4. Discussion and Limitations

**Figure 5:**
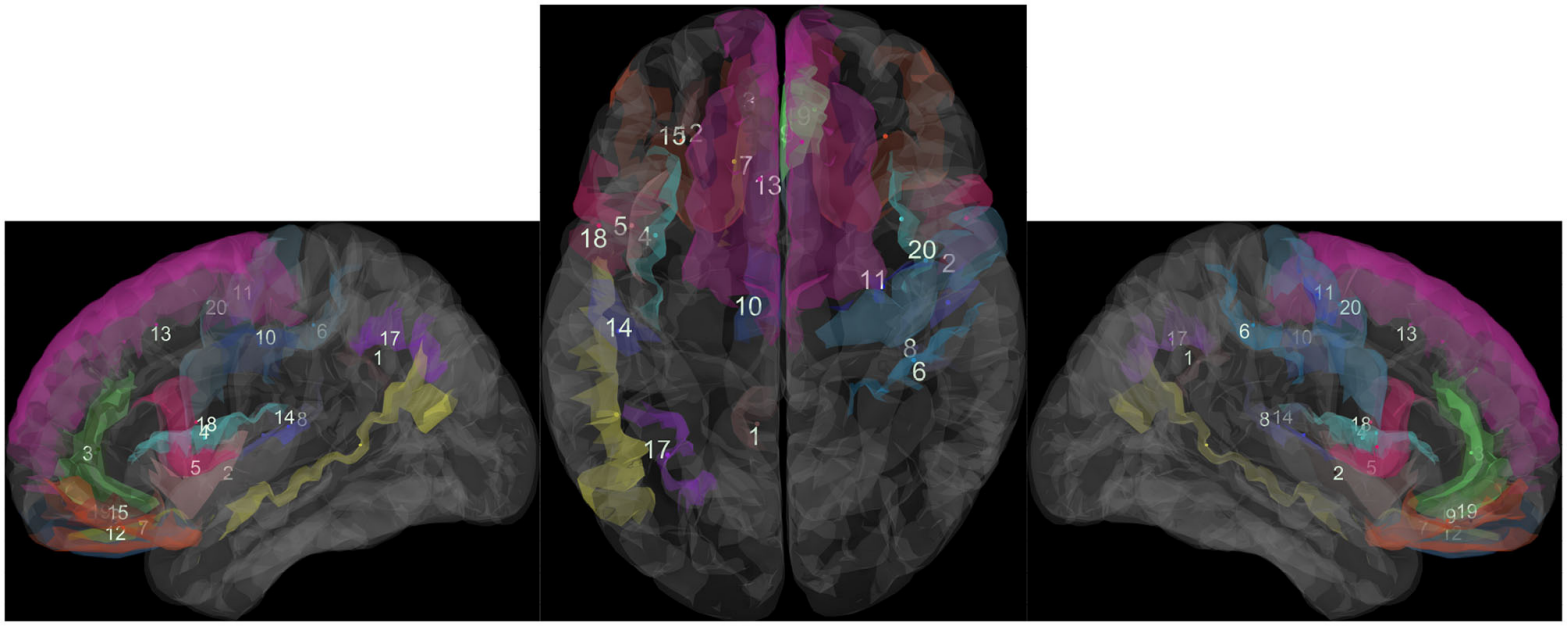
Brain areas most significant for predicting source credibility evaluations. Automatic parcellation of human cortical gyri and sulci using standard anatomical nomenclature. See Table 6.

Based on experimental results, we can distinguish areas of the brain that are most involved in source credibility evaluation: subparietal sulcus; insular gyrus and central insular sulcus; as well as anterior part of the cingulate gyrus and sulcus; circular sulcus of the insula superior. These areas are the most significant ones of all listed in Table 5, ordered by their significance for the logistic regression classifier of source credibility evaluations, based on MEC in the time interval from 500 to 750 ms from stimulus. Figure 5 presents a visualization of these brain areas.

**Table 5:** Brain areas, from which the Mean Electric Charge during the interval between 500 and 750 ms from stimulus was used as explanatory variables for the Logistic Regression Model of Source Credibility.

**Brain area**	**Logistic regression coefficient**
Subparietal sulcus L	0.002725181
Subparietal sulcus R	0.00267949
Long insular gyrus and central sulcus of the insula R	0.001669322
Middle-anterior part of the cingulate gyrus and sulcus (aMCC) L	0.001163805
Superior segment of the circular sulcus of the insula R	0.00113571
Short insular gyri L	0.000951506
Superior segment of the circular sulcus of the insula L	0.00092843
Postcentral sulcus R	0.000768929
Medial orbital sulcus (olfactory sulcus) L	0.000758103
Posterior ramus of the lateral sulcus R	0.000661928
Straight gyrus L	0.000641104
Middle-posterior part of the cingulate gyrus and sulcus (pMCC L	0.000626318
Superior part of the precentral sulcus R	0.000617
Orbital gyri L	0.000581156
Superior frontal sulcus	0.000580993
Anterior transverse temporal gyrus R	0.000567418
Orbital sulci L	0.000549164
Superior temporal sulcus L	0.000543894
Intraparietal sulcus and transverse parietal sulci L	0.000525286
Superior frontal gyrus (F1) L	0.000508378
Anterior part of the cingulate gyrus and sulcus (ACC) R	0.000489321
Opercular part of the inferior frontal gyrus R	0.000484836
Suborbital sulcus L	0.000463022
Straight gyrus R	0.000451529
Opercular part of the inferior frontal gyrus L	0.000439947
Orbital gyri R	0.000431694
Anterior transverse temporal gyrus L	0.000427869
Precentral gyrus R	0.000401498

*Brain areas are ordered by their decreasing significance for the model's prediction*.

The above mentioned areas were reported in the literature before, and they are more or less associated with the decision-making process (Pérez Álvarez and Timoneda Gallart, 2007; Lin et al., 2008; Stern et al., 2010; Uddin et al., 2017).

**Table 6:** List of the most influential brain areas. See the corresponding number in Figure 5.

**No**.	**List of anatomical parcellations**
1	Subparietal sulcus
2	Long insular gyrus and central sulcus of the insula
3	Anterior part of the cingulate gyrus and sulcus
4	Superior segment of the circular sulcus of the insula
5	Short insular gyri
6	Postcentral sulcus
7	Medial orbital sulcus
8	Posterior ramus of the lateral sulcus
9	Straight gyrus
10	Middle-posterior part of the cingulate gyrus and sulcus
11	Superior part of the precentral sulcus
12	Orbital gyri
13	Superior frontal gyrus
14	Anterior transverse temporal gyrus
15	Orbital sulci
16	Superior temporal sulcus
17	Intraparietal sulcus
18	Opercular part of the inferior frontal gyrus
19	Suborbital sulcus
20	Precentral gyrus

Subparietal sulcus in other nomenclature associated with the Brodmann areas 23 and 24 are reported in fMRI studies (Pérez Álvarez and Timoneda Gallart, 2007) as involved in emotionally engaged decision-making, and they are second-most active areas during the experiment conducted there. Participants had to make decisions under the influence of moral dilemmas.

Insular gyrus and central insular sulcus (sometimes simply called insula) are one of the least understood areas of the human brain. For our research, the most important of all its function seems to be the involvement in risky decision making (Harlé et al., 2012; Uddin et al., 2017). The “somatic marker” hypothesis assumes that emotions influence the decision process through internal sensations, visceral, and musculoskeletal physiological changes that are associated with reinforcing stimuli (Harlé et al., 2012; Uddin et al., 2017). Given its role in viscerosensory processing and its connections with the orbitofrontal cortex—a key structure in the decision-making circuitry—the insula is likely to play a critical role in risky decisions (Uddin et al., 2017). In our research, we have found another function of this brain area: that of source credibility evaluation.

Anterior part of the cingulate gyrus and sulcus is reported in Stern et al. (2010) to be also involved in the decision-making process under strong influence of uncertainty of conditions (Stern et al., 2010). In Brodmann areas nomenclature these are areas 32 and 24.

Circular sulcus of the insula superior is a special part of insula, found in Lin et al. (2008) to play an important role in long-term guidance of decision-making in one of the experiments that used the Iowa Gambling Task (IGT) (Lin et al., 2008).

The question is how the decision-making task in our experiment was associated with the level of source credibility? The point is that all decisions in our experiment were made at some level of uncertainty, which suggests that along with (Kawiak et al., 2020) our participants were always uncertain of the given response. Depending on SCL, the level of uncertainty changes. This change is visible in Figure 2 that illustrates hypothesis 1. So it is of no surprise that statistically significant differences were observed in the activity of anterior part of the cingulate gyrus and sulcus, and that this region is one of the most important for the machine classification model.

During the experiments, our participants probably tried to discover some decision rules or strategies. That supports the findings in Stern et al. (2010) where the IGT insula is also involved, as in the case of gambling and some other risky games (Wojcik et al., 2019).

But what about subparietal sulcus and emotions? Are there any emotions in assessing responses of students about which the participants do not even know? Emotional engagement in our experiment should be rather low. We postulate that subparietal sulcus is just a part of source credibility level evaluation circuitry and constitutes the foundation of *a credibility loop* in the brain. Orbital gyri (corresponding to the BA 10, 11, 12) could also be involved in the credibility loop. The process of credibility evaluation observed in our experiments is in many ways similar to the process of decision-making with moral dilemmas, judgement, and assessment of probability for likely and unlikely rewards.

We had designed the experiment of source credibility evaluation with consideration for internal validity. The choice of Japanese language (Kanji signs) translation was motivated by the desire to exclude confounding variables such as participant prior knowledge, experience or opinions on the subject of the evaluated message. We were also able to verify internal validity by evaluating the impact of the main independent variable (Source Credibility Level, SCL) on the participant's decisions (as stated by hypothesis 1. The experiment also had a 0% attrition rate, as all participants completed the experiment.

External validity of our experiment is difficult to establish, as it was one of the first experiments regarding brain activity during credibility evaluation reported in scientific literature. In order to increase its external validity, the experiment was designed to increase psychological realism and relevance for participants. In order to achieve this goal, we decided to use an exam or test setting that should be familiar for our participants who were university students. We can compare the results of this experiment to our pilot experiment (Kawiak et al., 2020). This experiment had a similar setting, but it also included avatars. The pilot experiment had a completely separate and smaller set of participants (57). Similar hypotheses were posed in the pilot experiment as in the experiment reported in this article, and their verification results are the same. The logistic regression model reported in Kawiak et al. (2020) cannot be compared to the model reported in this article. This is due to the fact that we have used Brodman areas in the analysis of the pilot experiment, while we are using brain areas in this article; another reason is that in the pilot experiment we have selected different time interval for analysis (150–600 ms from stimulus, while in this experiment the time interval is 500–750 ms). The two time intervals were chosen using a similar method, yet the pilot experiment had a slightly shorter duration, and EEG signals in the pilot experiment were estimated based on a smaller sample of participants. Overall, we consider that the preliminary results in the pilot experiment confirm the external validity of our experiment, because the results of hypothesis verification based on both experiments were the same.

Our experiment has several limitations. First, only right-handed, young men who were university students of a technical subject were included in our sample.

Second, our experiment controlled and limited the factors that could influence credibility evaluation. Only source credibility operationalized as the accuracy in the overall test (reputation in context of expertise) was available to experiment participants. While this setting resembled a situation in which a social media user evaluates the credibility of a message on an unfamiliar subjects, the experimental setting was still very limiting. Other factors, such as the source's gender, look, race, age or social status, could influence source credibility.

## 5. Conclusion and Future Work

The results of our experiment point to the existence of a brain credibility loop that ought to be investigated in future experiments. It could be useful to repeat the same experiment using fMRI equipment.

Except for the MRI studies, we intend to add some psychological characteristics of future participants. It could be very interesting to find whether there is a correlation between personality features and other psychological observables, and the brain processes involved in credibility evaluation, especially in the postulated credibility loop.

From the technical point of view, it would be useful to check the performance of other Machine Learning Tools in modeling credibility-oriented behavior registered using electrical activity of the brain. This task is especially important from the perspective of future applications that would require higher accuracy of predicting credibility evaluations.

One such possible application would be the credibility evaluation of debunking information designed to counteract fake news. The findings from this study can be used to guide the design of future experiments with a panel of judges who would evaluate the credibility of fake news or debunking information. In particular, the observation of the time interval (500–750 ms from stimulus) and most influential brain areas (see Table 6) can serve as a basis for designing a source credibility evaluation method. However, further experiments are needed to study other essential aspects of credibility, such as message credibility. Once the brain activity involved in making source and message credibility evaluations will be known, it would be possible to investigate the question: is fake news found credible because of its content, or because of the identity of the source (typically, a social media user who is a friend of the receiver)?

There are still many questions to be answered, for example: are our brains similar or different in the aspect of credibility evaluation? Would it be possible to build a universal model of brain activity and classifier able to measure its level with accuracy higher than 90%? Or—could it be better to generate individual models for particular participants?

Answering such questions require much more laboratory work and data science engineering. We would like to encourage researchers to work on this hitherto unexplored topic.

## Data Availability Statement

The raw data supporting the conclusions of this article will be made available by the authors, without undue reservation.

## Ethics Statement

The studies involving human participants were reviewed and approved by Bioethical Commission of Maria Curie-Sklodowska University in Lublin, Poland. The patients/participants provided their written informed consent to participate in this study.

## Author Contributions

All authors listed have made a substantial, direct and intellectual contribution to the work, and approved it for publication.

## Conflict of Interest

The authors declare that the research was conducted in the absence of any commercial or financial relationships that could be construed as a potential conflict of interest.

## Footnotes

^1^“How to fight an infodemic?,” The Lancet, February 19th, 2020. https://www.thelancet.com/journals/lancet/article/PIIS0140-6736(20)30461-X/fulltext.

^2^Conspiracy Theories Prosper During Coronavirus Pandemic, Snopes.com, accessed April, 21st 2020, https://www.snopes.com/news/2020/04/09/5g-conspiracy-theories-prosper-during-the-coronavirus-pandemic/.

^3^Electrical Geodesic Systems, Inc., 500 East 4th Ave. Suite 200, Eugene, OR 97401, USA.

^4^The electrodes are described in EGI 256-channel cap specification as best for cognitive ERP observations, covering the scalp regularly and numbered as follows: E98, E99, E100, E101, E108, E109, E110, E116, E117, E118, E119, E124, E125, E126, E127, E128, E129, E137, E138, E139, E140, E141, E149, E150, E151, E152. Those electrodes are automatically chosen for observing P-300 ERP signal by NetStation software.

^5^See: pubmed.ncbi.nlm.nih.gov/25907442/
